# HDAC1 and HDAC3 underlie dynamic H3K9 acetylation during embryonic neurogenesis and in schizophrenia-like animals

**DOI:** 10.1002/jcp.25914

**Published:** 2017-05-03

**Authors:** Josef Večeřa, Eva Bártová, Jana Krejčí, Soňa Legartová, Denisa Komůrková, Jana Rudá-Kučerová, Tibor Štark, Eva Dražanová, Tomáš Kašpárek, Alexandra Šulcová, Frank J. Dekker, Wiktor Szymanski, Christian Seiser, Georg Weitzer, Raphael Mechoulam, Vincenzo Micale, Stanislav Kozubek

**Affiliations:** 1 Faculty of Science, Department of Experimental Biology, Masaryk University, Brno, Czech Republic; 2 Institute of Biophysics of the Czech Academy of Sciences, v.v.i., Brno, Czech Republic; 3 Faculty of Medicine, Department of Pharmacology, Masaryk University, Brno, Czech Republic; 4 Institute of Scientific Instruments of the Czech Academy of Sciences, v.v.i., Brno, Czech Republic; 5 Behavioral and Social Neuroscience Group, CEITEC—Central European Institute of Technology, Masaryk University, Brno, Czech Republic; 6 Chemical and Pharmaceutical Biology, University of Groningen, Groningen, The Netherlands; 7 Department of Radiology, University of Groningen, University Medical Center Groningen, Groningen, The Netherlands; 8 Max F. Perutz Laboratories, Vienna Biocenter (VBC), Vienna, Austria; 9 Faculty of Medicine, Institute for Drug Research, Hebrew University of Jerusalem, Jerusalem, Israel; 10 Department of Biomedical and Biotechnological Sciences, Section of Pharmacology, School of Medicine, University of Catania, Catania, Italy

**Keywords:** acetylome, H3K9 acetylation, HDACs, mouse neurogenesis, schizophrenia

## Abstract

Although histone acetylation is one of the most widely studied epigenetic modifications, there is still a lack of information regarding how the acetylome is regulated during brain development and pathophysiological processes. We demonstrate that the embryonic brain (E15) is characterized by an increase in H3K9 acetylation as well as decreases in the levels of HDAC1 and HDAC3. Moreover, experimental induction of H3K9 hyperacetylation led to the overexpression of NCAM in the embryonic cortex and depletion of Sox2 in the subventricular ependyma, which mimicked the differentiation processes. Inducing differentiation in HDAC1-deficient mouse ESCs resulted in early H3K9 deacetylation, Sox2 downregulation, and enhanced astrogliogenesis, whereas neuro-differentiation was almost suppressed. Neuro-differentiation of (wt) ESCs was characterized by H3K9 hyperacetylation that was associated with HDAC1 and HDAC3 depletion. Conversely, the hippocampi of schizophrenia-like animals showed H3K9 deacetylation that was regulated by an increase in both HDAC1 and HDAC3. The hippocampi of schizophrenia-like brains that were treated with the cannabinoid receptor-1 inverse antagonist AM251 expressed H3K9ac at the level observed in normal brains. Together, the results indicate that co-regulation of H3K9ac by HDAC1 and HDAC3 is important to both embryonic brain development and neuro-differentiation as well as the pathophysiology of a schizophrenia-like phenotype.

## 1 Introduction

The histone code is responsible for maintaining higher-order chromatin structure and gene expression by mediating protein–protein and protein–DNA interactions. Histone acetylation and histone methylation represent basic epigenetic changes that regulate gene expression. Histone modifications have also been shown to regulate neural fate commitment in pluripotent stem cells via bivalent effects on both pluripotency and pro-neural genes. In human embryonic stem cells (ESCs), pluripotency genes that were highly transcriptionally active were associated with active histone modifications such as H3K9 acetylation (H3K9ac) (Krejci, Bernard, Housden, Collins, & Bray, 2009). Upon neural differentiation of ESCs, the genes that are not involved in this maturation pathway become inaccessible to transcription factors as a result of increased histone H3K27 methylation. Moreover, so-called bivalent domains that consist of H3K27 methylated regions that harbor smaller regions of H3K4 methylation regulate the silencing of developmental genes in ES cells (Bernstein et al., 2006). Despite these observations, the precise roles of specific histone modifications during neural development and especially the exact mechanisms by which histone acetylation is regulated during neurogenesis have not yet been fully elucidated.

The acetylation of histones represents a very prominent post-translational modification. The levels of this histone modification are balanced by the opposing functions of histone acetyl transferases (HATs) and histone deacetylases (HDACs) (Jenuwein & Allis, 2001; Kouzarides, 2007). One of the best-described characteristics of HDACs is the regulation of self-renewal and proliferation in neural stem cells (NSCs). The inhibition of HDACs often leads to neural differentiation (reviewed in Sun, Fu, Shen, & Shi, 2011). Most of the experimentally and clinically relevant HDAC inhibitors (HDACi), including valproate (VPA), trichostatin A (TSA), and suberoylanilide hydroxamic acid (SAHA), affect neuronal differentiation via histone hyperacetylation of transcription regulators and neurotrophic factors, which leads to their activation (Balasubramaniyan et al., 2006; Hsieh, Nakashima, Kuwabara, Mejia, & Gage, 2004; Siebzehnrubl et al., 2007). However, the effect of inhibitors is substantially pleiotropic, and only a few studies have focused on the specific role of HDACi in neurogenesis. Among the HDAC protein families, the class I deacetylases are particularly well-known as key regulators of neural development and differentiation (Sun et al., 2011). Embryonic neural stem/progenitor cells (NSPC) express high levels of HDAC1 whereas HDAC2 is upregulated during neurogenesis in post-mitotic neuroblasts and neurons (MacDonald & Roskams, 2008). HDAC1/2 have been observed to play similar roles in neurogenic zones in the adult brain, including the sub-ventricular zone (SVZ) and sub-granular zone (SGZ) of the hippocampus (Foti, Chou, Moll, & Roskams, 2013). Another class I deacetylase, HDAC3, has also been shown to be crucial for proper embryonic neural development in several studies. For example, dysfunctions in this deacetylase in the NSC pool is lethal during early development. In contrast, deleting HDAC3 later, in E15-derived NSCs, increased neurogenesis (Norwood, Franklin, Sharma, & D’Mello, 2014). Additionally, the absence of HDAC3 but not HDAC2 initiated neuronal differentiation pathways in NSCs (Castelo-Branco et al., 2014). With respect to the NSPCs in the adult brain, it was additionally observed that functional HDAC3 is responsible for cell proliferation and differentiation (Jiang & Hsieh, 2014). Therefore, it seems clear that the functions of specific HDACs are required for neural development, but their roles in this process are distinct in different developmental stages and in different regions of the brain.

Aberrant histone acetylation has also been reported in neurological diseases, including schizophrenia (Abdolmaleky & Thiagalingam, 2011). Schizophrenia is a complex disorder that is defined by psychotic, affective, and cognitive symptoms. Several causative factors have been found to be responsible for the onset of this illness. A substantial proportion of altered cognitive processes involve the dysfunction of the hippocampus and prefrontal cortex (PFC) during postnatal maturation and neuronal pruning (Antonova, Sharma, Morris, & Kumari, 2004; Shorter & Miller, 2015). Evidence for epigenetic abnormalities in this disorder comes from studies of schizophrenia candidate genes that have been shown to be responsible for regulation of neurotransmitter metabolism, cell adhesion, or neuronal growth (Guidotti et al., 2005; Lewis, Hashimoto, & Volk, 2005). In schizophrenia, elevated levels of methylation and decreased levels of acetylation of specific histones have often been found to be associated with the reduced expression of genes that regulate neuronal metabolism (Akbarian, 2010; Chase, Gavin, Guidotti, & Sharma, 2013; Gavin, Kartan, Chase, Jayaraman, & Sharma, 2009). The non-specific inhibition of HDACs has been shown to ameliorate cognitive phenotypes and to enhance learning and memory tasks in schizophrenia patients (Fischer, Sananbenesi, Wang, Dobbin, & Tsai, 2007). However, the pleiotropic activity not only of the HDAC inhibitors but also other antipsychotic drugs including the commonly used haloperidol has been associated with severe side-effects and is not always therapeutically potent in schizophrenia (Schwarz, Volz, Li, & Leucht, 2008). One attractive therapeutic target in many psychoses, including schizophrenia, is the endocannabinoid system. From this view, the most studied agent is the non-psychotropic phytocannabinoid, cannabidiol (Iseger & Bossong, 2015). This drug mainly acts as an indirect antagonist of cannabinoid signaling but also has a variety of side-effects with biological manifestation (Mechoulam, Peters, Murillo-Rodriguez, & Hanus, 2007; Pertwee, 2008). Recent papers have demonstrated that the pharmacological useof cannabidiol (CBD) prevents psychosis-like symptoms in experimental animal models and was also effective in clinical trials in schizophrenic patients (Iseger & Bossong, 2015). There are indications that above mentioned drugs can target the epigenome, especially with respect to DNA methylation and the expression of HDACs, but there is limited information regarding the potential direct effect of these drugs on histone acetylation in neurological diseases including schizophrenia (Abdolmaleky & Thiagalingam, 2011; Aberg et al., 2013; Boks, 2014; Melas et al., 2012).

Based on these observations, we designed experiments to explore the connections between the regulation of the acetylome and neural development in rodent embryonic and adult brains under both physiological and pathophysiological conditions. Using HDAC inhibitors and an HDAC1*-*knockout ES cell line, we demonstrated the existence of a regulatory link among HDAC1, HDAC3, and histone H3K9 acetylation in NSCs during both embryonic brain development and in vitro neural differentiation. We then focused on the regulation of the acetylome in the main neurogenic regions including the hippocampus and olfactory bulbs in a neurodevelopmental model of schizophrenia that is induced by prenatal administration of methylazoxymethanol acetate (MAM). In this schizophrenia-like model, our question was whether cannabinoid-based antipsychotic drugs and haloperidol have the potential to influence the protein levels to achieve levels similar to those observed in the control brains. We believe that our work increases the understanding of the complex epigenetic processes, particularly the acetylome in the regulation of brain development and during the manifestation of neurological diseases, including schizophrenia.

## 2 Results

### 2.1 In E15 brains, increased H3K9 acetylation and decreased HDAC1 and HDAC3 levels were accompanied by the loss of NSC markers and the onset of neurogenesis

**Figure 1:**
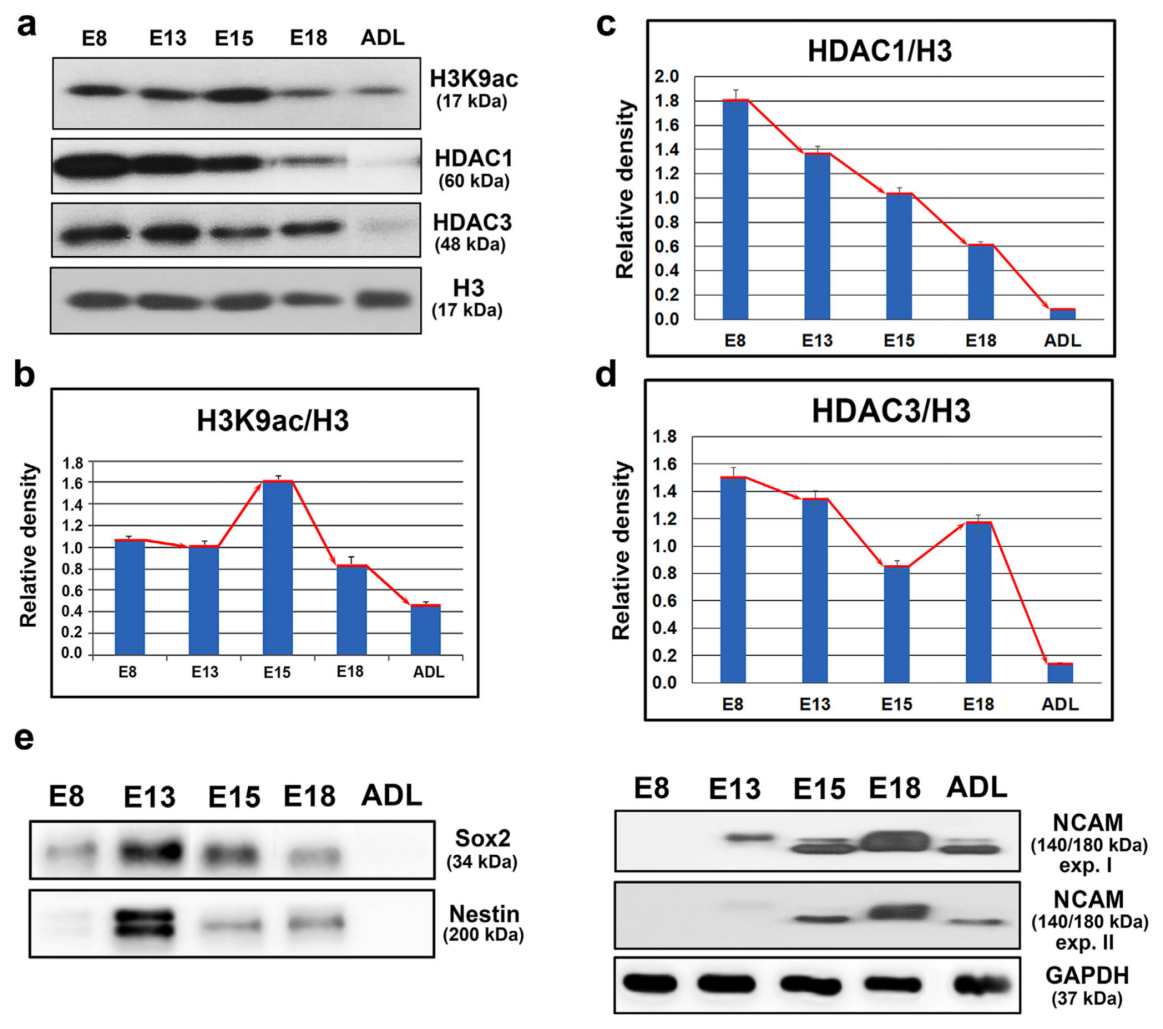
The levels of H3K9 acetylation, HDAC1, HDAC3, and neural markers in embryonic and adult mouse brains as determined using Western blot analysis. (a) Western blots showing the levels of H3K9ac, HDAC1, HDAC3, and total histone H3 in E8, E13, E15, and E18 mouse brains and adult brains (ADL). The levels of the histone acetylation-related modifications were quantified using ImageJ software, and the results were normalized to the histone H3 levels. The results are shown as graphs for (b) H3K9 acetylation, (c) HDAC1 levels, and (d) HDAC3 levels. The red line with arrows indicates the trend in protein levels. Panel (e) shows the levels of the Sox2, Nestin, and NCAM proteins in embryonic brains of E8, E13, E15, E18 stages, and in adult (ADL) mouse brain. Two Western blot images (expositions) are shown

We used Western blot analysis to evaluate the physiologically relevant dynamics in the acetylome of the developing brain using the brains of embryonic day 8 (E8), E13, E15, and E18 and adult mouse brains. The level of H3K9ac was nearly identical in E8 and E13 samples, but at E15, there was a substantial increase in H3K9ac that represented its highest acetylation level during mouse brain development. After this developmental point, the H3K9ac levels markedly decreased from E18 until adulthood (Figure 1a,b). The HDAC1 levels continuously decreased from E8 until adulthood, when it became nearly undetectable (Figure 1a,c). However, while the level of HDAC3 was high and stable from E8 until E13, a remarkable decline in the HDAC3 level was found at E15 before it increased again at E18. In the adult brains, HDAC3 was almost undetectable (Figure 1a,d). To explore the importance of the acetylome profile in neural development, we evaluated the levels of specific markers of neural differentiation in brain tissues (Figure 1e and quantification in S1A,a1–a3). The neuroepithelial and neural stem cell marker Sox2 was significantly expressed at E8, reached maximum expression at E13, similar to that of Nestin, and then continuously decreased until E18 (Figures 1e, S1Aa, and Ab). The expression of NCAM, a marker of early neuronal differentiation and migration, increased from E13 to E18: the highest level was found at the E18 stage (Figures 1e, S1Ac). These observations demonstrate that at E15, a decrease in NSC markers and pronounced neural differentiation was accompanied by H3K9 hyperacetylation and decreases in the levels of HDAC1 and HDAC3.

### 2.2 Chemical inhibition of HDACs caused histone hyperacetylation in E15 brains

**Figure 2:**
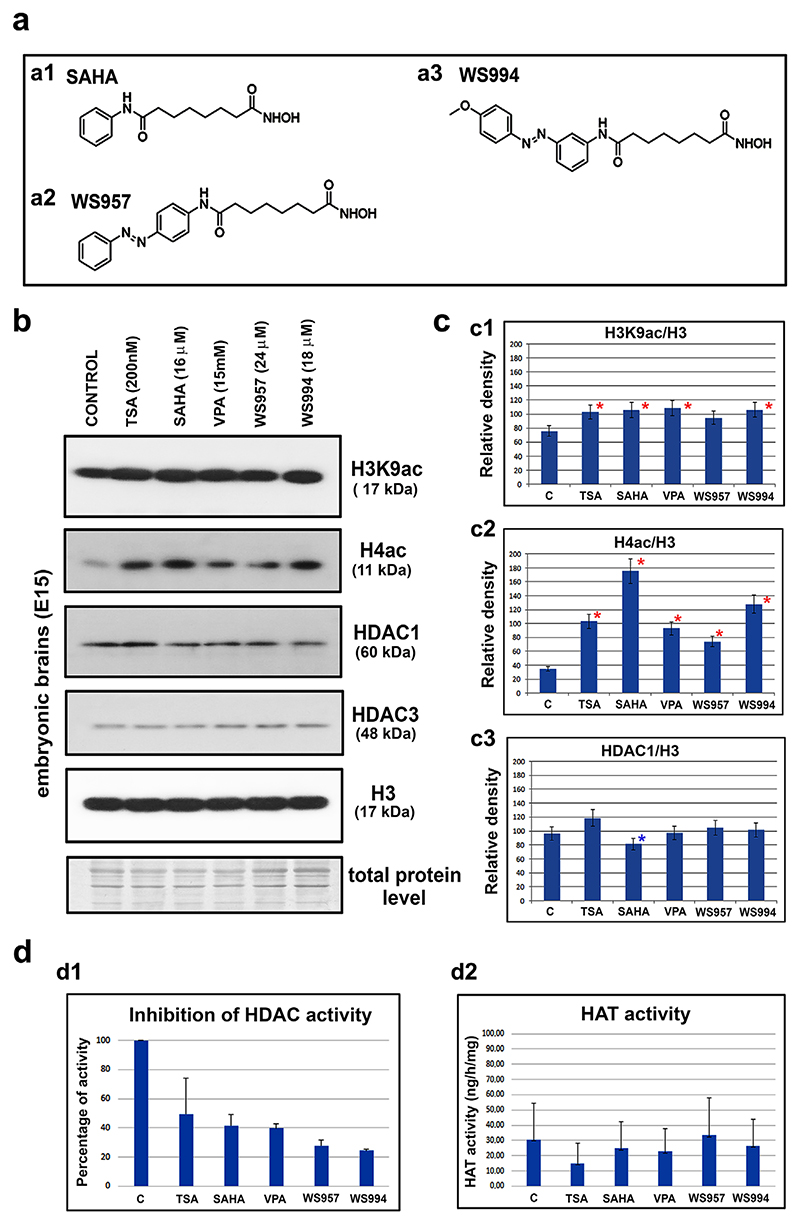
The epigenetic patterns were examined in embryonic and adult mouse brains using Western blot analysis. The effects of the HDAC inhibitors TSA and SAHA and two new photo-switchable SAHA analogs (WS957 and WS994) were tested. (a) The formulas of (a1) SAHA, (a2) WS957, and (a3) WS994. (b) Western blot analysis was performed to determine the levels of H3K9ac, H4ac, HDAC1, HDAC3, and total histone H3 in the following samples: control non-treated E15 brains and E15 brains treated with TSA, SAHA, VPA, WS957, or WS994. (c) The quantification of the data shown in panel b showing the normalized results of the Western blot data to demonstrate the levels of (c1) H3K9ac, (c2) H4ac, (c3) HDAC1 with reference to the level of total histone H3. The bar charts show the means ± standard errors. The quantification of the protein levels was performed using ImageJ software. (d) The total (d1) HDAC activity (an inhibition of HDACs activity is shown) and (d2) HAT activity was evaluated after treatment of the E15 brains with the various HDAC inhibitors

We next established an experimental model to determine whether we could manipulate the acetylome and mimic the events that characterize neurogenesis during embryonic development. Figure 2 shows the effects of treatment with selected HDAC inhibitors (HDACi) on the epigenome at the time that the highest level of H3K9ac was observed (E15 stage; see Figure 1a). We tested whether H3K9 hyperacetylation in E15 brains was potentiated by HDACi and whether the level of H3K9ac was regulated by HDAC1 or HDAC3 and/or by both HDACs.

In explanted brains obtained from E15 embryos, we induced H3K9 and H4 hyperacetylation using synthetic HDAC inhibitors at pharmacologically relevant concentrations (Figure 2a,b). We studied the effects of TSA, SAHA (syn. Vorinostat), valproate (VPA), and the newly synthesized photo-switchable derivatives of SAHA (compounds WS957 and WS994, which are azobenzene-bearing derivatives of SAHA, Figure 2a,a1–a3; [Szymanski, Ourailidou, Velema, Dekker, & Feringa, 2015]). We found that all of the HDAC inhibitors had the ability to increase H3K9ac in explanted brains 3 hr after treatment. SAHA was the most powerful inducer of histone hyperacetylation (Figure 2b,c,c1,c2). Although the HDAC3 levels remained stable after treatment with all of the tested HDACis (Figure 2b), among the tested chemical compounds, only SAHA displayed the potential to significant decrease HDAC1 levels (Figure 2b,c,c3).

Surprisingly, the TSA-treated brains were characterized by a slight increase in the level of HDAC1 (Figure 2c,c3). Based on this observation, we performed additional tests of the HDAC and HAT activity (Figure 2d, d1,d2). We observed a pronounced inhibition of the HDAC activity by all of the HDAC inhibitors tested (Figure 2d,d1). The most pronounced inhibitory effect was found after the WS957 and WS994 treatments. However, the levels of the HATs were not affected by HDACi treatment in explanted mouse brains (Figure 2d,d2).

Together, our experiments showed that SAHA was the most potent inhibitor of HDAC1. For these reasons, and because SAHA has been shown to be a therapeutically promising HDACi in clinical trials, we tested the effects of this drug in our next experiments. We also analyzed the effect of TSA, which has been shown to be a potent regulator of neuronal differentiation in embryonic brains (Shaked et al., 2008). The results showed that the various HDAC inhibitors substantially induced (at selected concentrations) histone hyperacetylation in E15 brains but had no direct effect on the HATs.

### 2.3 Levels of Sox2 were decreased and neural differentiation was enhanced after experimentally induced histone hyperacetylation in E15 brains

**Figure 3:**
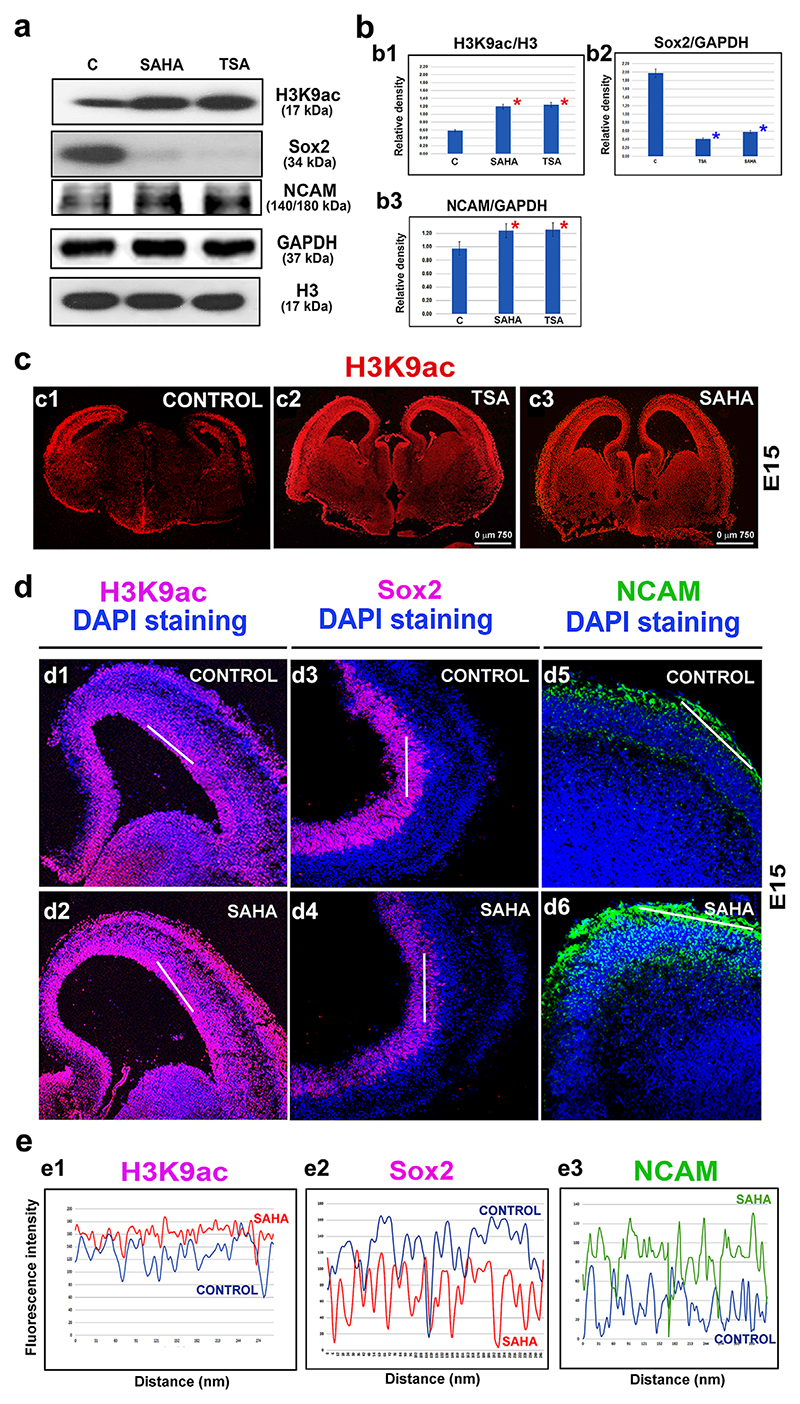
Studies of H3K9ac and neural markers in E15 mouse brain explants that were treated with HDACi. The following proteins were evaluated using Western blot analysis: H3K9ac, Sox2, and NCAM in control, non-treated E15 brains and in brains that were treated with SAHA and TSA. (a) The Western blot images show representative data from 3 to 5 independent biological replicates. The Western blot images differ in Figures 1a and 3a to properly show the differences in the experimental outcomes. The E15 embryonic stage was characterized by a very high level of H3K9 acetylation, and this stage was selected for the testing of the HDAC inhibitors as shown in Figure 3a. Panel (b) shows the quantification for (b1) H3K9ac, (b2) Sox2, and (b3) NCAM. The densities of the bands were analyzed using ImageJ software, and the protein levels were evaluated following normalization to the level of GAPDH or histone H3 in the case of H3K9ac. The red asterisks show statistically significant differences in the protein levels. Panel (c) documents a representative E15 brain cryosection labeled to reveal H3K9 acetylation (red) in (c1) non-treated, (c2) TSA-treated, and (c3) SAHA-treated brains. (d) A high level of H3K9ac was observed in the ventricular ependyma in (d1) non-treated and (d2) SAHA-treated brains explants. The level of Sox2 was high in (d3) the ventricular ependymal but was decreased (d4) after treatment with SAHA. NCAM was expressed at the cortex periphery in (d5) non-treated brains, and its expression was higher (d6) after treatment with SAHA. DAPI was used to visualize the DNA contents of the tissues. (e) The levels of H3K9ac (white lines) were quantified using ImageJ software (see d1–d6 in panel d). Panel (e) shows the comparison between the protein levels observed in the control non-treated and SAHA-treated E15 brains: (e1) H3K9ac, (e2) Sox2, and (e3) NCAM

To determine whether hyperacetylation can induce neural differentiation similar to that observed during development, we analyzed the neuro-differentiation in E15 brains after treatment with TSA or SAHA. In parallel with the increases in the H3K9ac levels that were observed after treatment with either HDACi, the level of the NSC marker Sox2 was markedly decreased whereas the NCAM level was increased (Figure 3a,b,b1–b3). These results resembled those observed at the E15 stage during development. Moreover, the differences in the protein levels were even more pronounced by exposure to the HDACis than the changes observed in vivo (Figures 1e, 3a).

Immunostaining of coronal sections of the brains showed that H3K9ac was prominently localized in the ventricular ependyma (VE), the ganglionic eminence (GE), and the marginal zone of the cortex (Figure 3c, c1,d,d1,e,e1). After treatment with the HDACis, the H3K9 hyperacetylation was ubiquitously observed in all of these zones (Figure 3c,c2, c3). After treatment with SAHA, the Sox2 expression was downregulated in parallel with an increase in H3K9 acetylation in the VE (Figure 3c,c3,d,d3,4e,e2). Concomitantly, NCAM was upregulated in parallel with SAHA-increased acetylation in the marginal zone of the cortex (Figure 3d,d5,d6,e,e3). These results clearly demonstrate that hyperacetylation that was induced by the selected HDACis strengthened the neuronal differentiation in the E15 brain explants and that these effects resembled the developmental changes that were observed at the E15 stage during physiological development of the brain.

### 2.4 An increase in the H3K9ac levels in neuro-differentiated wild type mESCs was accompanied by decreases in the HDAC1 and HDAC3 levels whereas a lineage shift toward astrogliogenesis that was characterized by H3K9 deacetylation was induced in differentiated HDAC1 knockout mESCs

**Figure 4:**
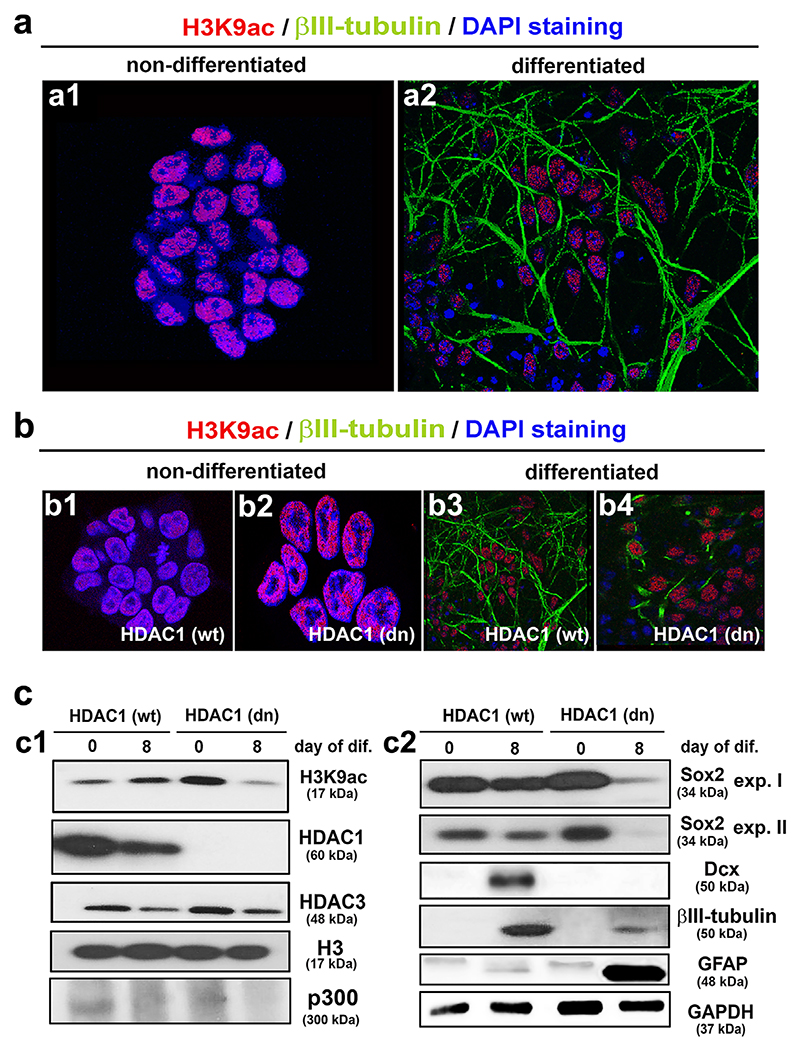
The distribution pattern of H3K9ac and the levels of histone-related markers in HDAC1 (wt) D3 mESCs and HDAC1 (dn) control mouse embryonic stem cells (mESCs) in cells undergoing neural differentiation. (a) H3K9ac (red) is shown in (a1) non-differentiated (wt) D3 mESCs and (a2) cells undergoing differentiation as indicated by βIII-tubulin positivity (green). (b) H3K9ac (red) and βIII-tubulin (green) in (b1) non-differentiated (wt) mESCs, (b2) non-differentiated HDAC1 (dn) mESCs, (b3) differentiated (wt) mESCs, or (b4) differentiated HDAC1 (dn) mESCs. DAPI (blue) was used to counterstain the cell nuclei. (c) Western blot analysis revealed the levels of (c1) H3K9ac, HDAC1, HDAC3, histone H3,and p300 or (c2) the neuronal marker Sox2 (two images of different expositions), Dcx, βIII-tubulin, and GFAP in non-differentiated and differentiated (wt) D3 mESCs and HDAC1 (dn) mESCs. GAPDH was used as the reference protein for the neural markers, and histone H3 was used as the reference for markers of the acetylome

From our previous experiments and data in the literature, HDAC1 appears to be a crucial deacetylase in the regulation of neural differentiation in the brain. To investigate this phenomenon more specifically, we used an HDAC1 double-knockout (dn) embryonic stem cell line to mimic neurogenesis in vitro. Mouse embryonic stem cells (mESCs) were differentiated for 8 days in serum-free medium supplemented with *all-trans* retinoic acid (ATRA). H3K9 acetylation was most prominently detected in the cells present in the center of the mESC colony whereas neuronal differentiation was induced mainly in peripheral cells that did not contain H3K9ac but were positive for βIII-tubulin (Figures 4a,a1,a2; S1B). In both wt and knock-out cells, the differentiation process was also accompanied by a dramatic reduction in cell size that indicated chromatin condensation, which is a sign of differentiation-related transcriptional inactivation (Figures 4b; S1Ca, b). In addition, on the 4th day of differentiation, we observed an increase in the H3K9 acetylation in the wt cells, and a modest decrease in this histone modification was observed from the 8th until the 12th day of cell maturation (S1Da). In the HDAC1 (dn) cells, a high H3K9ac level was found in the undifferentiated mESCs, which remained high until the 4th day of differentiation and then decreased from the 8th to the 10th day as shown by Western blot analysis (Figure S1Db). In the cell nuclei, immunocytochemistry confirmed that non-differentiated HDAC1 (dn) mESCs expressed a much higher level of H3K9 acetylation than was observed in the HDAC1 (wt) controls (Figure 4b,b1,b2). However, on the 8th day of neural differentiation, the H3K9ac levels were substantially lower in the HDAC1 (dn) cells, as shown by both Western blot analysis and immunocytochemistry (Figures 4b,b3,b4, c,c1, S1Db). In the wt cells, neural differentiation resulted in lower levels of HDAC1/3 and a slightly higher level of H3K9 acetylation than those observed in the non-differentiated counterpart (Figures 4c,c1, S2A–C). To determine whether the changes in H3K9 acetylation during cell differentiation (in Figure 4c,c1) were a consequence of HDAC1 depletion or a consequence of HAT activation, we further analyzed the p300 histone acetyltransferase. We observed that the level of p300 was relatively high in the (wt) ESCs and non-differentiated HDAC1 (dn) ESCs. However, in parallel with the H3K9 deacetylation in the HDAC1 (dn) cells, the level of p300 HAT was decreased (Figure 4c,c1). Thus, in this case, the regulatory function with respect to H3K9ac could be attributed to p300.

Differentiated wt cells were also positive for early neuronal (doublecortin, βIII-tubulin) and a low level of the astroglial marker (GFAP) (Figure 4b,b3,c,c2). In contrast, the differentiated HDAC1-deficient cells displayed higher levels of HDAC3 compared to the differentiated wt cells as well as a marked decrease in the H3K9 acetylation (Figures 4c,c1, S2A). Interestingly, the neuronal marker doublecortin (Dcx) was not observed in the differentiated HDAC1 (dn) cells (Figure 4c,c2), and βIII-tubulin was barely detected in these cells (Figure 4b,b4,c,c2). The differentiated HDAC1 (dn) cells were characterized by abnormally high levels of the astroglial marker protein GFAP (Figure 4c,c2). Moreover, the NSC marker Sox2 was expressed in the differentiated wt cells but instead vanished during differentiation of the mutant cell line (Figures 4c,c2, S1Da, b). These data demonstrate that in wt ESCs, on the 8th day of differentiation, the acetylome and profiles of neuronal markers were highly similar to the epigenetic and differentiation profiles in the E15 brains (compare Figures 1a,e, and 4c,c1,c2, S2A–C). We also showed that the loss of HDAC1 resulted in early H3K9 deacetylation that accompanied astrogliogenesis and was additionally characterized by a relatively high HDAC3 level (S2A–C). Interestingly, the NSC pool was only depleted in the differentiated mutant cell line, which showed that NSCs underwent a lineage-specific shift toward astrogliogensis at the expense of neurogenesis.

**Figure 5:**
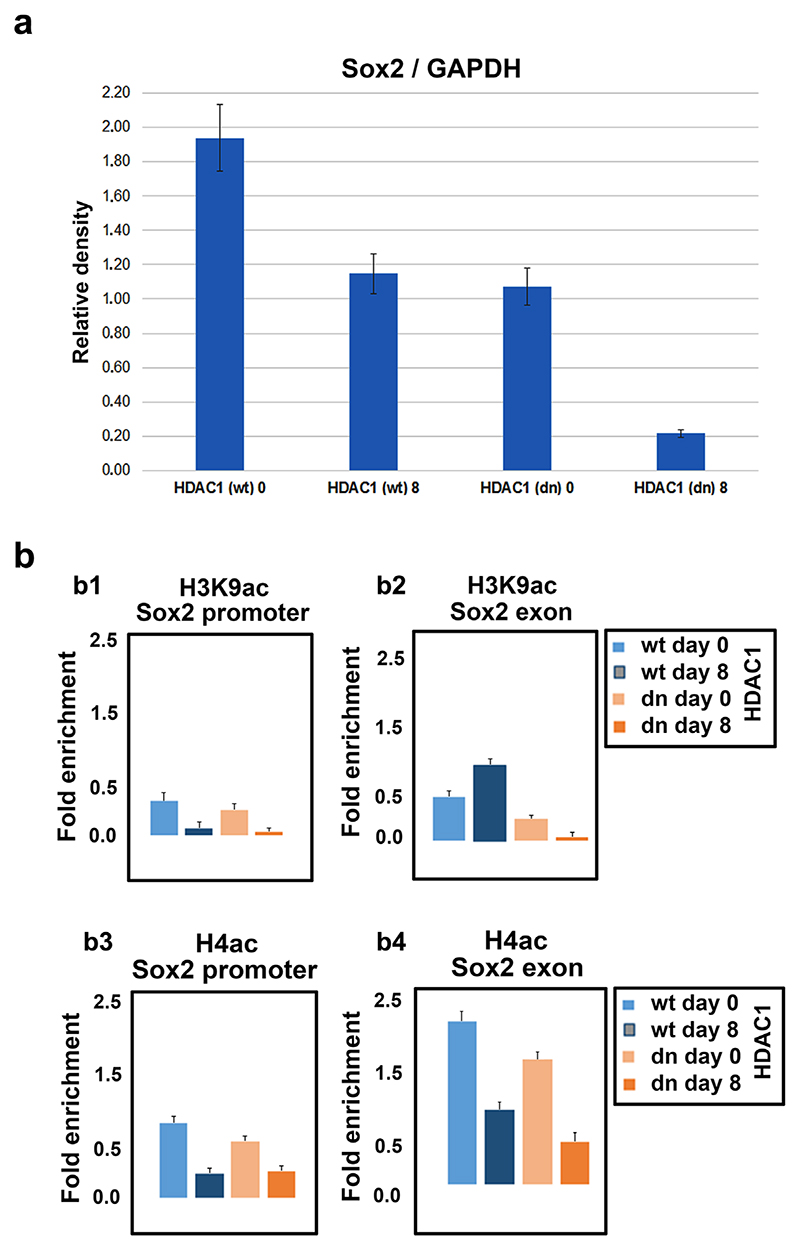
ChIP-qPCR analysis of H3K9ac and H4ac associated with the promoter and exon of Sox2 gene. (a) The level of Sox2 protein in Figure 4c,c2 was quantified using ImageJ software. The bar charts show the mean values ± standard errors calculated from three biological and nine technical replicates. (b) The levels of H3K9ac in (b1) the Sox2 promoter and (b2) the Sox2 exon were studied using ChIP-qPCR. The total H4ac in (b3) Sox2 promoter or (b4) Sox2 exon is shown

In addition, we used ChIP-PCR to study the levels of H3K9ac and H4ac associated with the promoter and exon of the Sox2 gene. In the promoter of Sox2, we observed a decrease in H3K9ac and H4ac in both differentiated HDAC1 (wt) and HDAC1 (dn) mESCs. This observation correlated with a pronounced decrease in the level of the Sox2 protein during the differentiation events (Figure 5a,b,b1,b3). On the other hand, in the Sox2 exon, although the H4 acetylation was systematically depleted in both differentiated (wt) and HDAC1 (dn) mESCs, H3K9ac was not depleted in the differentiated (wt) cells (Figure 5b,b2,b4). Thus, these experiments showed that H4ac, rather than H3K9ac, drives the Sox2 regulation during the differentiation processes, including neuro-differentiation and astrogliogenesis.

### 2.5 In the schizophrenia-like brain, treatment with specific antipsychotic drugs restored the levels of HDACs and H3K9 acetylation to those observed in normal brains

**Figure 6:**
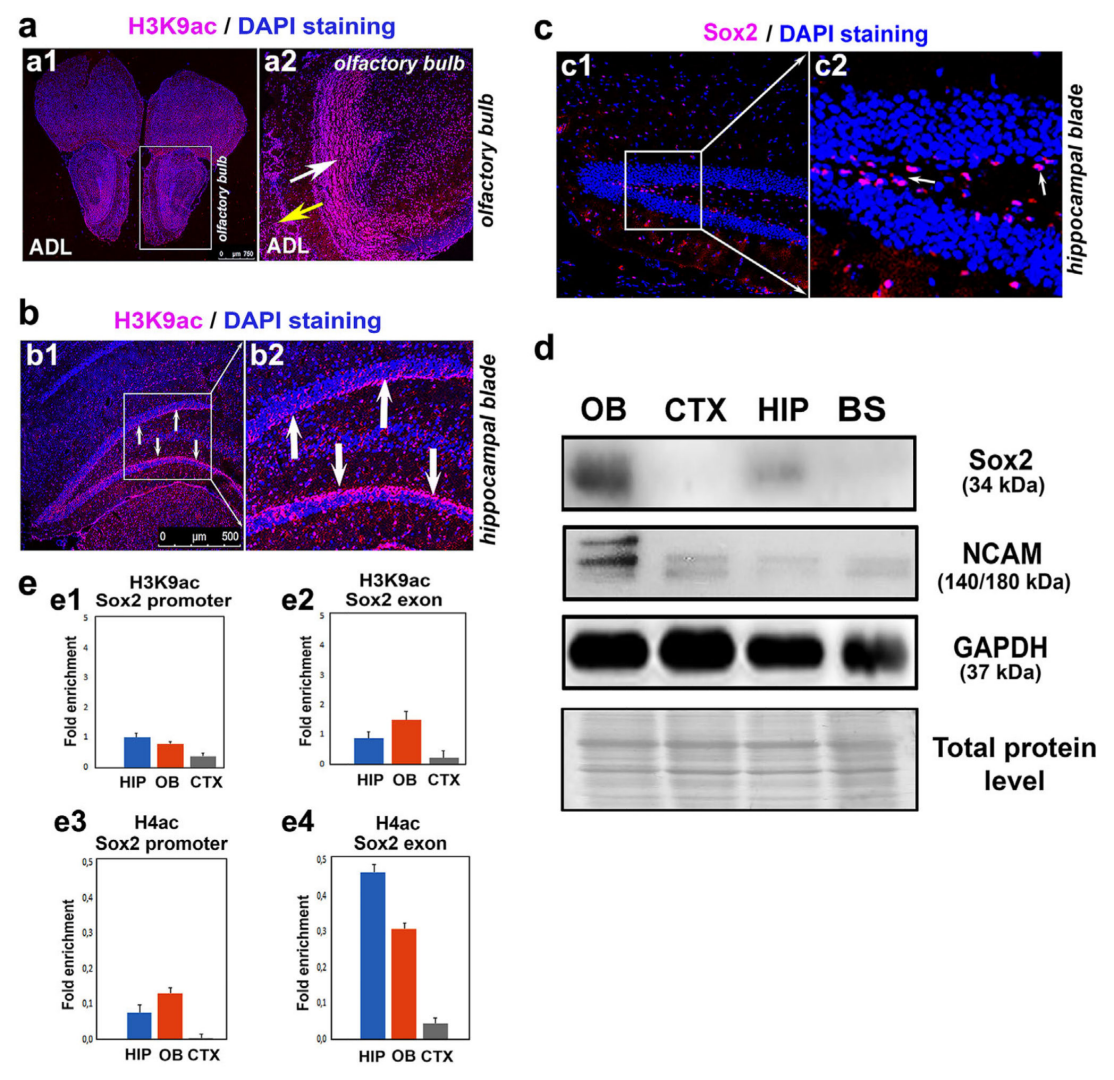
H3K9 acetylation in the olfactory bulbs and hippocampal regions of adult mice. (a) H3K9 acetylation in (a1) an entire adult brain section and (a2) the olfactory bulb in an adult brain. The white arrow indicates the granular layer, and the yellow arrow indicates the glomerular layer of the adult olfactory bulb. (b) H3K9 acetylation (pink) in (b1) the hippocampal blade of the mouse hippocampus, and (b2) a magnification of the frame outlined in panel (b1). (c) The Sox2 (pink) distribution pattern in (c1) the mouse hippocampal blade, and (c2) a magnification of the frame outlined in panel (c1). The arrows shown in (cc2) indicate the Sox2-positive cell nuclei. (d) Western blot analysis revealed the levels of Sox2, NCAM, and GAPDH in isolated adult olfactory bulbs (OB), the cortex (CTX), the hippocampus (HIP) and brain stem (BS). (e) In the OB, HIP, and CTX, the levels of H3K9ac in (e1) the Sox2 promoter and (e2) the Sox2 exon and H4ac in (e3) the Sox2 promoter or (e4) the Sox2 exon were studied by ChIP-qPCR

As the first step in assessing the effects of antipsychotic drugs, we determined the pattern of distribution of H3K9 acetylation in the neurogenic zones of normal adult mouse brains. We mainly focused on the olfactory bulbs (OBs) and hippocampal region, both of which are known to be zones of active neurogenesis and which are also the most prominently altered brain regions in schizophrenia (Aberg et al., 2013). These regions are therefore, potential targets for treatments that involve the administration of known antipsychotic drugs and innovative treatment options. We found that the cells of the granular layer of the OBs and the dentate gyrus of the hippocampus were characterized by high levels of H3K9 acetylation, which was also a consequence of a high density of cell nuclei (Figure 6a,a1,a2; b,b1,b2). This acetylation state was accompanied by a relatively high level of expression of Sox2 and NCAM, especially in the olfactory bulbs compared with the cortex (CTX) or brain stem (Figure 6c,c1,c2; 6d, S2D). We therefore, confirmed that in the adult brain, hyperacetylation, in combination with Sox2 positivity, preferentially occurs in regions that are undergoing active neurogenesis. In this context, we studied the levels of H3K9ac and H4ac associated with the promoter and exon of the Sox2 gene (Figure 6e,e1–e4). Compared to the CTX, the olfactory bulbs of the adult mouse brain, which had high levels of Sox2, were characterized by a higher level of H3K9ac in the Sox2 exon and elevated H4ac in both the promoter and exon of Sox2 (Figure 6d,e, e1–e4). These data emphasize that H4ac is a regulatory factor that is especially associated with Sox2 over-expression (Figure 6d,e,e1–e4).

**Figure 7:**
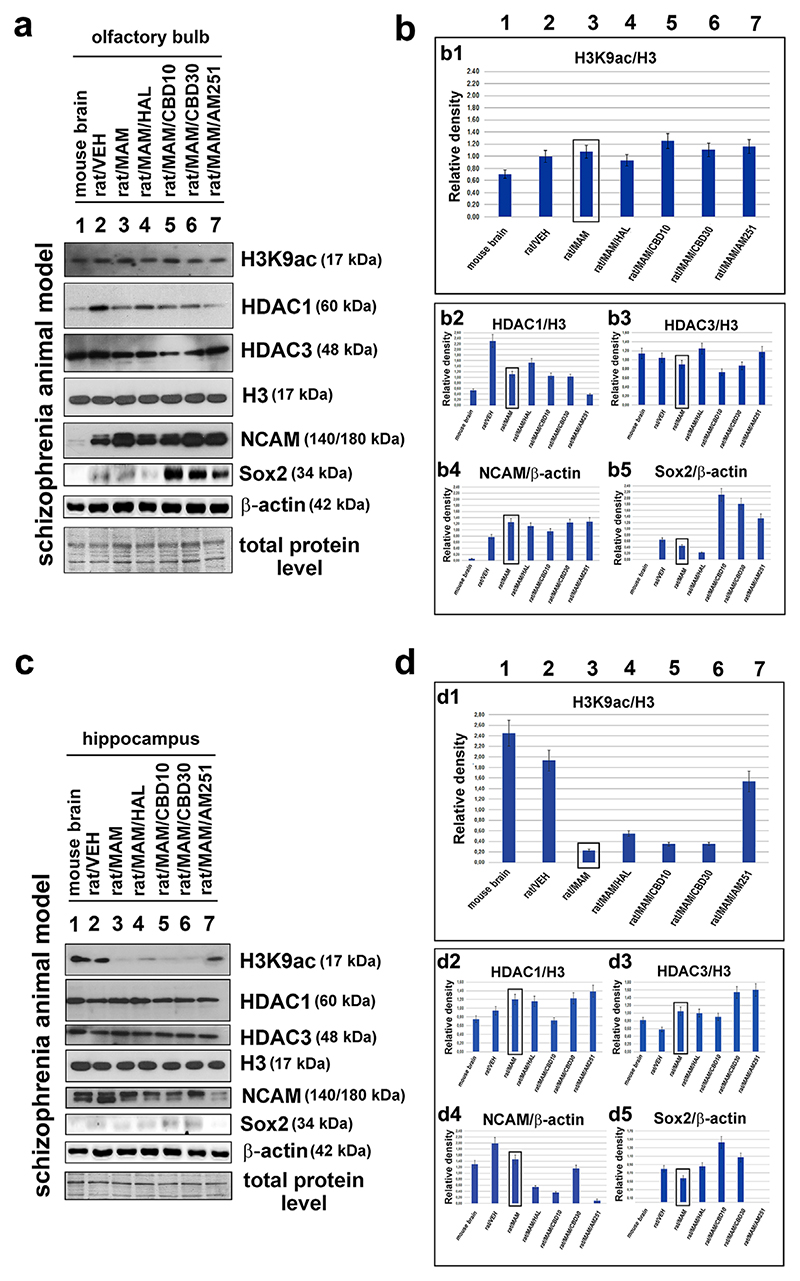
The epigenetics of the olfactory bulb and the hippocampus in control and schizophrenia-like rats. Schizophrenia was pharmacologically induced, and the resulting schizophrenia-like rats (labeled MAM) were treated with anti-psychotic drugs. The following proteins were studied using Western blot analysis: H3K9ac, HDAC1, HDAC3, total H3, NCAM, Sox2, and β-actin. The effects of the following anti-psychotic drugs were tested: haloperidol, cannabidiol CBD10, CBD30 (two doses), and AM251. Panel (a) shows the original Western blot data for samples that were isolated from the olfactory bulbs. The protein loading was based on identical levels of total proteins. The quantification was performed using ImageJ, and Western blot bands were normalized to the total histone H3 levels (or to β-actin for the neural markers), as shown in panel (b) for (b1) H3K9ac, (b2) HDAC1, (b3) HDAC3, (b4) NCAM, and (b5) Sox2. The Sox2 level was normalized not only to the level of β-actin but also to the total protein level used for Western blot loading. After data normalization, the Sox2 level was decreased in the MAM samples compared to physiological brains. Panel (c) shows the original Western blot data for samples that were isolated from the hippocampi of the examined rats. (d) The quantification of levels of (d1) H3K9ac, (d2) HDAC1, (d3) HDAC3, (d4) NCAM, and (d5) Sox2. The analysis was performed using normal mouse and rat brains and brains explanted from schizophrenia-like rats that were treated with haloperidol, cannabidiol, CBD10/30 and AM251. For each Western blot lysate, we used one OB and one hippocampal region. The analysis was performed using three biological replicates

In the next experimental step, using a rat model of schizophrenia induced by prenatal administration of MAM, we studied the acetylome and neural status in the neurogenic zones, including OBs and hippocampus. For these analyses, we selected schizophrenia-like rats because recently this experimental model was fully established in our laboratory and represent a useful tool for studies of this neurological disorder (Jones, Watson, & Fone, 2011; Ruda-Kucerova et al., 2016). In the OBs, we observed that the level of H3K9 acetylation was slightly higher in the MAM animals and that the levels of both HDAC1 and HDAC3 were lower, particularly for HDAC1 (Figure 7a,b,b1–b3). Both isoforms of NCAM (140 and 180 kDa) were expressed at markedly higher levels whereas Sox2 was downregulated in the OBs of the MAM animals (Figure 7a,b,b4,b5). In the hippocampus, the neuro-epigenetic status of the tissue was different: the H3K9 acetylation in the MAM animals dropped to almost undetectable levels, whereas both HDAC1 and HDAC3 were slightly upregulated as shown by the quantitative analysis of Western blots (Figure 7c,d,d1–d3). The 140 kDa isoform of NCAM decreased in the hippocampus of the MAM rats whereas the level of 180 kDa isoform was high (Figure 7c,d,d4). Sox2 was even more depleted in the MAM model animals than in the controls, and overall, the Sox2 protein was barely detectable in hippocampi of the schizophrenia-like animals (Figure 7c,d,d5).

**Figure 8:**
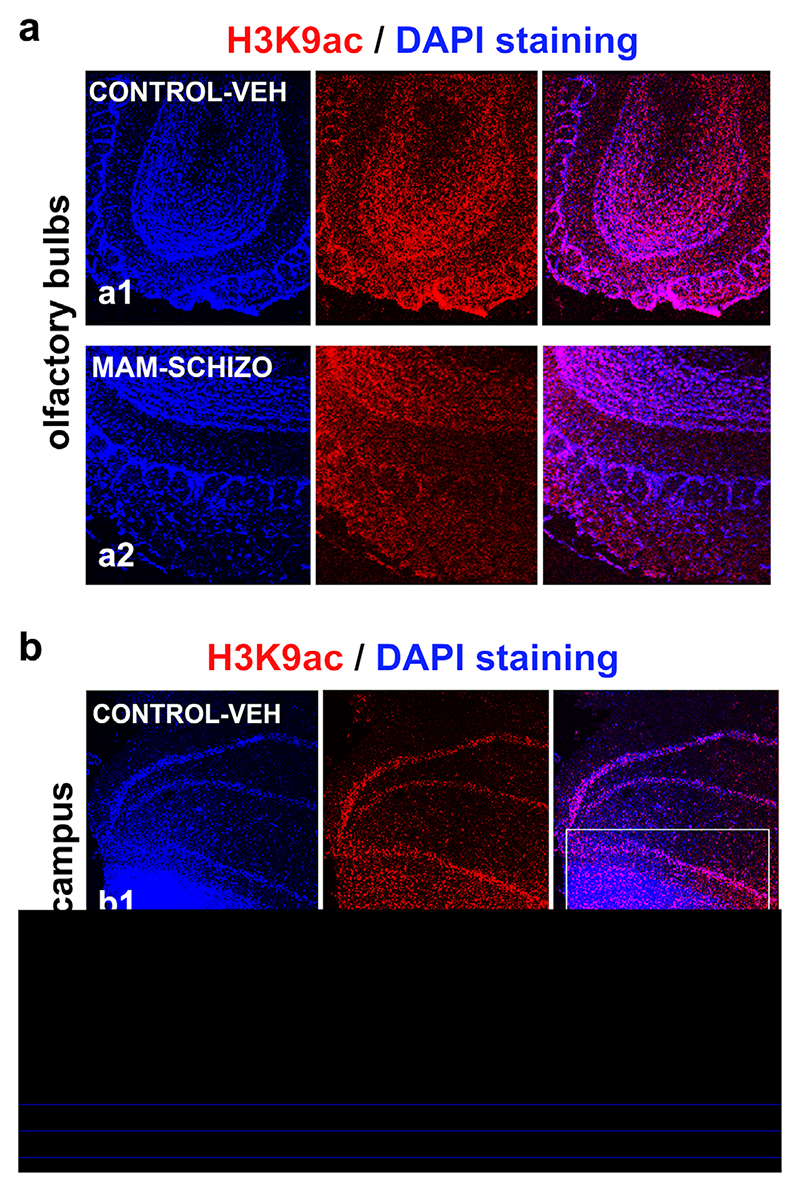
The levels of H3K9 acetylation in the rat hippocampal regions and olfactory bulbs were studied using immunocytochemistry. (a) H3K9ac in the olfactory bulbs of (a1) control non-treated animals (vehicle; VEH) and (a2) schizophrenia-like animals (labeled MAM). (b) H3K9ac in hippocampus of (b1) control non-treated animals (vehicle; VEH) and (b2) schizophrenia-like animals (labeled MAM). The white frames in panels bb1, b2 show a reduced level of H3K9ac in intra-pyramidal blade of granule cell layer of hippocampus in the schizophrenia-like experimental model compared to the control non-treated animals

Compared to the olfactory bulbs with stable H3K9ac in control and schizophrenia-like brains (Figure 8a), a lower level of H3K9ac was demonstrated by immunocytochemistry in the hippocampi of the schizophrenia-like animals (Figure 8b). A similar trend for H3K9ac was shown in the Western blots (Figure 7a–d). In the MAM animals, immunocytochemistry showed H3K9 deacetylation in intra-pyramidal blade of granule cell layer of hippocampus (Figure 8b,b1,b2).

Next, we tested the effects of haloperidol and experimental cannabinoids. Both the acetylome and the neural changes observed in the OBs of MAM animals were corrected to the patterns observed in the normal brains after treatment with haloperidol (Figure 7a,b). However, the schizophrenia-like traits in the hippocampus were somewhat suppressed by cannabidiol (CBD) or AM251, a cannabinoid receptor-1 inverse agonist (Figure 7c,d). The most remarkable result was the recovery-inducing effect of AM251 on H3K9 acetylation (Figure 7c,d,d1). Both HDAC1 and HDAC3 were susceptible to the effects of the dose of CBD used in the experiment, 10 mg/kg (Figure 7c,d,d2,d3). Both CBD and AM251 also effectively reduced the NCAM levels in the hippocampus, although the effect targeted the 180 kDa isoform but not the 140 kDa variant (see Figure 7c for both isoforms separately and Figure 7d,d4 for the simultaneous quantification of the isoforms). Additionally, CBD was found to be a very potent activator of the Sox2 protein, especially in the OBs (Figure 7a,b,b5). Taken together, the pathological changes in the acetylome and neural markers showed that the hippocampus and OBs displayed distinct characteristics, and drugs that modulated the cannabinoid signaling likely drove the recovery of these changes to physiological levels via different molecular mechanisms and pharmacological effects.

## 3 Discussion

Class I HDACs have often been shown to be associated with brain development, as documented by many authors (Norwood et al., 2014; Volmar & Wahlestedt, 2015). In our studies, we focused on embryonic brains at various developmental stages to study the dynamics of histone acetylation. We chose to explore stages E8 to E18 because the brain undergoes many important developmental changes during this period, including the transformation of neuroepithelial cells into populations of radial glia, the formation of intermediate progenitors, and the initiation of early neuronal differentiation and migration (Zou et al., 2010). We observed the most striking changes in the acetylome around E15. At this developmental stage, we found a significant drop in the HDAC3 protein levels that was associated with an increase in the H3K9ac levels (Figure 1a,b,d). Unlike HDAC3, the level of HDAC1 continuously declined from E8 to E18, and this decrease was correlated with increases in the levels of the early neuronal marker NCAM and the depletion of the NSC marker Sox2 (Figure 1c–e). These data implied that HDAC1 and HDAC3 are both necessary for the regulation of H3K9ac in the embryonic brain (Figure 1a–d). It was recently reported that in NSCs derived from E15 brains, HDAC3 occupies the promoters of the neuronal/oligodendrocyte-related genes and that the absence of HDAC3 initiates neuronal differentiation via H3K9 hyperacetylation (Castelo-Branco et al., 2014). Another study claimed that HDAC1 is expressed in the NSCs and that as neuronal progenitors differentiate and the number of post-mitotic neurons increases during brain development, HDAC1 is replaced by HDAC2 (MacDonald & Roskams, 2008). However, deletion of either HDAC1 or HDAC3 during embryonic development results in either a severe neurophenotype or early lethality at approximately the E9 stage (Lagger et al., 2002; Montgomery et al., 2008; Norwood et al., 2014).

We additionally observed that HDAC1-deficient mESCs displayed an abnormal peak in H3K9 hyperacetylation that was followed by the sudden deacetylation and depletion of the NSC pool as the cells differentiated (Figures 4c,c1,c2, S2A). The neural differentiation of the HDAC1-deficient mESCs was associated with disrupted neurogenesis and an abnormal increase in astrogliogenesis as indicated by a substantial overexpression of GFAP (Figure 4c,c2). It seems apparent that the optimal function of HDAC1 during neural differentiation is crucial and irreplaceable for the neuronal populations to be properly established. However, we also observed that the HDAC3 level was high and the glial population was augmented in the differentiated HDAC1-deficient cells (Figures 4c,c1,c2, S2C). In 2008, Shaked et al. (2008) showed that in E15 brains, general inhibition of HDACs resulted in an increase in the production of immature astrocytes. Nevertheless, HDAC3 was recently reported to activate oligodendroglial lineage-specific genes while suppressing the genes that are responsible for astroglial differentiation (Zhang et al., 2016). HDAC3 also: (i) regulates the genes that drive neural differentiation in mESC-derived NSCs and (ii) has a strong and selective effect on post-mitotic neurons (Bardai & D’Mello, 2011; Castelo-Branco et al., 2014). Together, these results showed that the functions of both HDAC1 and HDAC3 are necessary for the optimal balance between neuronal and glial development in the embryonic brain and that depletion of these enzymes can result in neural phenotypes associated with abnormal function.

A few hours after chemical inhibition of the HDACs with TSA and SAHA, we observed H3K9 hyperacetylation in E15 brain explants. However, the Western blots did not show a remarkable inhibitory effect on the selected HDACs (HDAC1/3), with the exception that SAHA suppressed HDAC1 (Figure 2b,c). On the other hand, evaluation of the global HDAC activity showed an inhibitory effect of all of the studied HDAC inhibitors that induced H3K9 and H4 hyperacetylation in E15 brain explants (Figure 2d,d1,d2). Moreover, these pharmacologically induced histone acetylation changes were accompanied by a decrease in the Sox2 levels in the ventricular ependyma and an increase in neuronal differentiation marker NCAM in the embryonic cortex, as shown for SAHA treatment (Figure 3a,b,b1,b2; d,d3–d6). These data are interesting from a pharmacological point of view because we provided evidence using brain explants that experimental use of these agents induced a change in the histone signature within hours (Figure 3a,c,c1–c3; d,d1,d2; e,e1). Thus, this type of experiment suggests a method for testing the effects of promising epi-drugs as the first step of an analysis that precedes the more invasive pharmacological tests on experimental animals. Furthermore, the effects of the HDACi observed in our experimental model were similar to those reported by Shaked et al. (2008), who demonstrated that treating E15 brains with TSA results in a decrease in neurogenesis in the ganglionic eminence but a modest activation of neurogenesis in the cortex. Here, we additionally showed a correlation between a high level of Sox2, H3K9 hyperacetylation, and differentiation events when HDAC1 was downregulated or depleted even though the HDAC3 function was not completely abolished (Figures 1a–d, stage E15; 4cc1, c2, see HDAC1 (dn) pluripotent ESCs and summary in 9a). It is interesting that during the differentiation of the HDAC1 (wt) ESCs, the level of HDAC1 was slightly reduced and H3K9ac was increased, but Sox2 was not downregulated. This cellular event was associated with neurogenesis (Figures 1a–d, 4c,c1,c2). However, when HDAC1 was completely depleted, H3K9ac was reduced and Sox2 protein was barely detected after differentiation was induced. Under these conditions, pronounced astrogliogenesis was observed at the expense of neurogenesis, and this event was likely mediated via HDAC3, the level of which was relatively high in this case (Figures 4c,c1,c2, and 9a).

**Figure 9:**
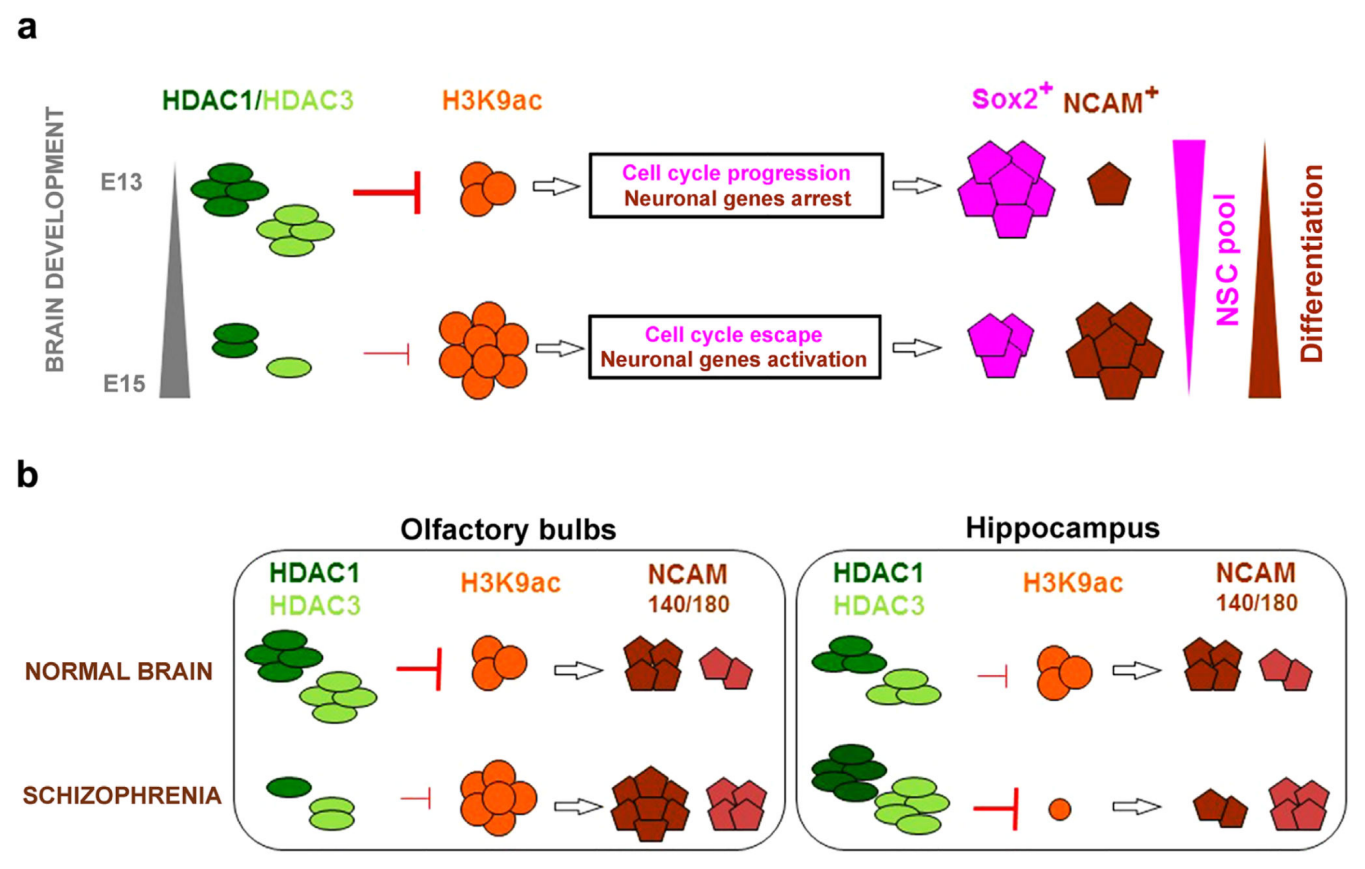
The regulation of the acetylome during embryonic brain development and in schizophrenia: a hypothetical model. (a) At E13, both HDAC1 and HDAC3 were highly expressed in the populations of NSCs in the brain, where they blocked the acetylation of histones at the promoters of transcription factors that are associated with neuronal and oligodendrocyte differentiation (Castelo-Branco et al., 2014; MacDonald & Roskams, 2008). Active HDAC1 and HDAC3 also maintain the proliferation and the non-differentiated status of the NSCs (Jiang & Hsieh, 2014; Lagger et al., 2002). The observation that the Sox2 levels were high demonstrated that the E13 brains were rich in NSCs. At E15, the low levels of both HDAC1 and HDAC3 resulted in an increase in H3K9 acetylation and the presence of loose chromatin at pro-neuronal genes. These changes were accompanied by an increase in the population of young neurons in the brain that were positive for NCAM whereas the Sox2-rich population of NSCs gradually disappeared. (b) In the olfactory bulbs of the schizophrenic brains, the HDAC1 and HDAC3 levels were low, but the levels of H3K9 acetylation and both NCAM isoforms were pathologically increased. In contrast, the hippocampi of the schizophrenia-like animals showed increased levels of HDAC1 and HDAC3, the absence of detectable H3K9 acetylation and an increase in the NCAM-180 isoform

Here, we also focused, from a pathophysiological point of view, on acetylome of schizophrenia-like experimental model. We observed that the hippocampi of schizophrenia-like rats had very low levels of H3K9 acetylation but displayed increases in both HDAC1 and HDAC3 levels (Figure 7c, d,d1–d3). In contrast, the HDAC1 levels were markedly lower in the OBs in the MAM model than in those of normal brains. This change was followed by a slight increase in the H3K9 acetylation (Figure 7a,b,b1–b3). Several pathological processes that lead to psychiatric disorders such as depression or schizophrenia have been associated with changes in histone acetylation and H3K4 methylation (summarized by [Huang et al., 2007; Ma et al., 2010; Shen, Shulha, Weng, & Akbarian, 2014]). In schizophrenia, the elevated methylation of histones, especially in the prefrontal cortex, is often associated with a decrease in the expression of genes that regulate neuronal functions and metabolism (Akbarian et al., 1995; Grayson et al., 2005). Interestingly, increased acetylation of histone H3 was observed in patients with bipolar disease (Gavin et al., 2009), whereas some schizophrenia patients were found to demonstrate lower levels of histone H3 acetylation (Gavin, Kartan, Chase, Grayson, & Sharma, 2008; Sharma et al., 2006). Non-specific inhibition of the HDACs was followed by an increase in H3 acetylation, which ameliorated the neurodegenerative and cognitive phenotypes in animal model of schizophrenia (Fischer et al., 2007). More specifically, in multiple postmortem cohorts of schizophrenic subjects, the expression of HDAC1 was found to be increased in the prefrontal cortex and hippocampus of the brain (Benes et al., 2007; Sharma, Grayson, & Gavin, 2008). We observed similar trends in the acetylome dynamics in the hippocampi of MAM rats, in that HDAC1 and HDAC3 were upregulated whereas H3K9 acetylation was nearly absent (Figures 7c, d,d1–d3, and 9b). In contrast, inducing the si-RNA-mediated knockdown of HDAC1 or the pharmacological inhibition of HDAC1 impaired learning and increased cell death (Kim et al., 2008), and these results correspond rather well with our observations in the OBs, in which schizophrenia-like phenotype led to the depletion of HDAC1 (Figure 7a,b,b2). These data emphasize the concept that schizophrenia is a complex pathology manifested in specific parts of the brain, and the hippocampus and olfactory bulbs could be candidates for these important regions (see summary in Figure 9b).

With respect to the neuronal markers, we found that the MAM rats displayed increased levels of NCAM in the OB whereas in the hippocampus, the increase was specific to the NCAM-180 isoform (Figure 7a,c). NCAM is one of many candidate molecules that has been associated with schizophrenic features such as ventricular enlargement, cognitive dysfunction, and putative neurodevelopmental etiologies (Vawter et al., 2000). Altered concentrations of the NCAM isoforms (120, 140, and 180 kDa) have been frequently reported in the hippocampus and prefrontal cortex (PFC) of schizophrenic patients (Gray, Dean, Kronsbein, Robinson, & Scarr, 2010; Vawter et al., 2001). However, there are discrepancies among the reports from several authors who have described pathological changes in the individual NCAM isoforms in schizophrenia. For example, NCAM-180 was found to be increased in schizophrenic patients (Gibbons, Thomas, & Dean, 2009; Tanaka, Yoshida, Shimada, Ueda, & Asai, 2007), but the cleaved NCAM isoform (cNCAM) was also observed in the hippocampus and PFC of schizophrenic brains (Honer et al., 1997; Vawter et al., 1998). Our results therefore, extend the previous evidence by showing that the NCAM-140 and NCAM-180 isoforms are altered in this model of schizophrenia, especially in the hippocampus and OBs, although it remains unclear whether NCAM plays a causal role in the development of schizophrenia symptoms in humans. Here, we also hypothesized that the increase in NCAM in the MAM animals was directly affected by the inhibition of HDACs, which led to an increase in the acetylation (Figure 9b). We clearly demonstrated that NCAM positivity was increased in the explanted E15 brains that were treated with the HDAC inhibitor SAHA (Figure 3a,b,b3; d,d5,d6; e,e3). Moreover, Lampen, Grimaldi, and Nau, (2005) showed that the inhibition of HDACs enhanced the expression of the NCAM mRNA and protein as well as the level of polysialylated NCAM (PSA-NCAM), a form of this protein that is primarily associated with neuronal migration but has also been associated with pathological phenotypes including schizophrenia. The increased polysialylation of NCAM was observed in autistic-like mice that were treated with several HDAC inhibitors including SAHA, and this alteration was accompanied by the complete recovery of the behavioral characteristics to a state similar to that observed in healthy adults (Foley et al., 2012). Hence, NCAM appears to be another important candidate molecule that is highly dynamic during both normal neural development and during pathophysiological processes and that is regulated by changes in the histone acetylome.

Brain psychiatric disorders can be cured using various psychotropic drugs. We tested some of these drugs in this study using a highly translational neurodevelopmental model of schizophrenia (Figure 7a–d). It has been shown, for example, that haloperidol and clozapine each affect the epigenome (Elia et al., 2014). Haloperidol decreases DNA methylation (Bertran-Gonzalez et al., 2009), and clozapine enhances the acetylation of promoter-associated H3K9, and H3K14 (Dong, Nelson, Grayson, Costa, & Guidotti, 2008). These results provide preliminary information to explain what happens to histone signatures or DNA methylation during therapy for neurological disorders such as schizophrenia. Furthermore, extremely limited information is available regarding the effects of cannabinoids on the histone signatures. Several recent studies have shown that some histone modifications are candidate targets for these drugs because a specific histone code is likely to underlie neurological functions and neurological diseases (DiNieri et al., 2011; Yang et al., 2014; Zumbrun, Sido, Nagarkatti, & Nagarkatti, 2015). Specifically, we showed here that the schizophrenia-like phenotype is characterized by acetylation-related patterns distinct from those of normal brains. However, in the MAM experimental model, some acetylation changes could be restored to a normal physiological state by cannabidinoid, which are therefore, potential candidates for so-called neuro-epidrugs (Figure 7a–d).

In conclusion, the general state of knowledge regarding the epigenetic processes in embryonic and adult brains remains at the level of initial studies, but enormous progress is being made. In neuropsychiatric diseases, the acetylation of histones by HATs and the functions of HDACs are the most frequently explored epigenetic features (Dean, Thomas, Lai, Chen, & Scarr, 2015; Grayson, Kundakovic, & Sharma, 2010). It is therefore, important to use animal models in which epigenetic markers undergo changes during brain development to determine whether histone acetylation alone is the important event that underlies developmental processes in the brain. From this perspective, studies that explore mechanisms that involve synergy between histone acetylation and individual HDACs might be of interest to neuroscientists in the future. Moreover, from the perspective of studies of brain regeneration, it is important to determine which brain regions and which developmentally important markers are subject to dynamic changes. This is especially important to know from the point of view of understanding the mechanisms by which clinically promising epi-drugs and other anti-psychotic drugs modulate the histone signatures. This knowledge provides a basis for our understanding of many neurological disorders and epigenetic treatments for them. This is an issue of a high importance in modern medicine.

## 4 Materials and Methods

### 4.1 Mice used for developmental studies

The C57/BL6 mice were obtained from the Breeding Facility of the Medical Faculty, Masaryk University, Brno, Czech Republic. The mice were housed in the Institute of Biophysics ASCR, v.v.i., in a Specific Pathogen-Free (SPF) animal facility, at a constant temperature of 21°C and 60% humidity under regular light/dark (12/12 hr) conditions. All experiments with mice were performed with the approval of the Ethics Commission of the Czech Academy of Sciences, v.v.i., protocol No.: 11/2015. The mice were sacrificed by overexposure to anesthetics (Narcamon/Rometar solution, Spofa, Czech Republic). Embryonic brains were analyzed on day 8.5, 13.5, 15.5, and 18.5 post-conception (here, these stages are designated E8, E13, E15, and E18 for simplicity).

### 4.2 Culture of HDAC1 double-null mouse embryonic stem cells and the induction of their differentiation

To perform the Western blot analyses and immunofluorescence experiments, we cultured D3 wild type (wt) mouse embryonic stem cells and HDAC1 (dn) mESCs as previously described (Lagger et al., 2002; Zupkovitz et al., 2010). The cells were cultured in DMEM (Gibco, Grand Island, NY) culture medium containing 15% fetal bovine serum, 0.1 mM non-essential amino acids, 100 µM MTG, 1 ng/ml LIF, 10,000 IU/ml penicillin, and 10,000 µg/ml streptomycin. The culture dishes were coated with Matrigel (#354277, Corning, Bedford, MA) according to the manufacturer’s instructions see also (Franek et al., 2015). The ES cells were seeded at 5,000 cells per cm^2^ in culture dishes. To induce differentiation, the mESCs were cultured without LIF. After 2 days, the culture medium was replaced with serum-free commercial medium DMEM/F-12 (1:1) (Gibco) supplemented with insulin, transferrin, and selenium (ITS-100x, Gibco), 1 μg/ml fibronectin according to (Pachernik, Esner, Bryja, Dvorak, & Hampl, 2002) (Sigma–Aldrich, Czech Republic), and a penicillin/streptomycin mixture. Neural differentiation was induced by adding 0.5 μM all-trans retinoic acid to the medium on the 3–4th day of differentiation (Sigma–Aldrich). On the 4th day of differentiation, the cells were washed three times with PBS, and the medium was changed every second day until days 8–16.

### 4.3 Chemical inhibition of histone deacetylases

Explanted E15 mouse brains were treated with the following HDAC inhibitors for 3 hr: 200 nM Trichostatin A (TSA; Sigma-Aldrich, represented by Cayman Pharma. s r.o., Czech Republic), 16 μM suberoylanilide hydroxamic acid (SAHA; Cayman Chemicals), 15 mM valproic acid (VPA) dissolved in ddH_2_0, or the photo-switchable analogs of SAHA, WS957 (24 μM) or WS994 (18 μM) (Szymanski et al., 2015). Except for VPA, the HDACi were dissolved in DMSO and adjusted to the final concentration in DMEM medium supplemented with 10% fetal calf serum (FCS). Then, the brain tissues were treated with the selected HDACi for 3 hr, and the samples were then lysed for Western blot analysis or fixed in 4% formaldehyde for immunohistochemical analysis. The effects of HDACi were also tested in mouse embryonic fibroblasts (MEFs) that were treated with 100 nm TSA (Sigma-Aldrich) or 8 μM SAHA (Cayman Chemicals), and these served as the positive controls for acetylation levels. The activities of the HATs and HDACs was studied using the EpiQuick HAT Activity/Inhibition Assay kit (Epigentek, represented by Epigentek, represented by Lab Mark, Czech Republic, #P-4003-48) and EpiQuick HDAC Activity/Inhibition Assay kit (Epigentek, represented by Lab Mark, #P-4002-48). These assays were performed according to the manufacturer’s instructions.

### 4.4 Rat model of schizophrenia and treatment schedules

All experiments were conducted in accordance with all relevant laws of the Czech Republic and all animal care and welfare regulations. The experimental protocol was approved by the Animal Care Committee of Masaryk University, Faculty of Medicine, Czech Republic, and the Central Ethical Committee of the Ministry of Education, Czech Republic and carried out according to the European Community guidelines for the use of experimental animals.

Pregnant female albino Sprague–Dawley rats were obtained from Charles River (Germany) at gestational day (GD) 13 and housed individually at the Breeding Facility of the Medical Faculty, Masaryk University, Brno, the Czech Republic. Each dam was randomly assigned to an experimental group, and on GD17, they were injected intraperitoneally (i.p.,) with either methylazoxymethanol acetate to induce the schizophrenia-like phenotype (MAM, Midwest Research Institute, Kansas City) (MAM, *n* = 3 rats) or vehicle (saline solution) (*n* = 3) as previously described (Moore, Jentsch, Ghajarnia, Geyer, & Grace, 2006; Perez, Shah, Asher, & Lodge, 2013). The MAM experimental model was optimized as previously described (Ruda-Kucerova et al., 2016). The mean litter size in the vehicle-treated mothers was 9.6 pups, and the mean litter size in the MAM-treated mothers was 11.5 pups. Only two litters were lost (killed by the mother): one control and one MAM-treated. The mothers were regularly weighed, and no difference was observed between the two experimental groups. The offspring were weaned on postnatal day (PND) 22 and housed in groups of two to three littermates until adulthood, at which time they were used in the behavioral and neurochemical experiments. Food and water were provided ad libitum, and the animals were housed under the following constant environmental conditions: relative humidity 50–60%, temperature 23°C ± 1°C, and an inverted 12-hr light-dark cycle (darkness from 7 a.m. to 7 p.m.). Only male offspring were used in this study.

All studied compounds were administered i.p. in a volume of 5 ml/kg body weight. An inverse agonist of the CB1 cannabinoid receptor, AM251 (Sigma-Aldrich), was dissolved in dimethyl sulfoxide (DMSO), Tween 80 and saline solution (1:1:8). The non-psychotropic experimental cannabinoid, cannabidiol, was obtained from the Hebrew University, Jerusalem, Israel. The compound was dissolved in Tween 80 (2%) and saline solution (98%). The typical antipsychotic drug haloperidol (Haloperidol-Richter®, Richter Gedeon, Czech Republic) was dissolved in saline solution and used as a positive control. Separate groups of the control animals and MAM-animals were injected i.p. with AM251, cannabidiol, or the haloperidol vehicle. The brains of the control groups were not included in the final study on the basis that the goal of the study was to determine which antipsychotic drugs could restore the studied proteins to levels similar to those observed in control brains (labeled VEH in Figure 6a–d). Therefore, only the MAM model and non-treated control brains were analyzed. To address our hypothesis, it was not necessary to know more about the effect of antipsychotic drugs on histone signature in the control (VEH)-treated animals.

From PND 19 to PND 39 (where puberty has been defined as PND 43.6 + 1 in Sprague–Dawley rats [Korenbrot, Huhtaniemi, & Weiner, 1977; Clark et al., 1999]), each group of rats (*n* = 3/per control group and *n* = 3/per MAM group) was treated i.p. with cannabidiol (CBD 10 or 30 mg/kg/day), AM251 (0.5 mg/kg/day), haloperidol (0.6 mg/kg/day), or vehicle based on methods described in previous studies (Gomes et al., 2015; Ruda-Kucerova et al., 2016; Valenti, Cifelli, Gill, & Grace, 2011; Valvassori et al., 2014; Zamberletti, Rubino, et al., 2012; Zamberletti, Vigano, et al., 2012). Using Western blotting, we evaluated two brains, and similar results we observed in both samples from each group. Moreover, four hemispheres were inspected. The third brain was used for verification of Western blot results by quantitative immunofluorescence (not shown). To avoid litter effects, each experimental group consisted of animals chosen randomly from different litters. The rats showed normal increases in body weight (measured every day from PND 19 to PND 39 and every other day from PND 40 to PND 85) independent of which prenatal treatment they received (i.e., MAM or saline solution) or whether the drug was administered preadolescence (data not shown). Adult animals (PND 90-PND 120) were subjected to behavioral tests. After the completion of the behavioral tests, the rats were immediately decapitated under inhalation anesthesia at the age of 20 weeks. The whole brains were obtained and fixed for analysis by Western blots and immunofluorescence. The brain of one animal from each group was dissected to separate the hemispheres and olfactory bulbs of the brain. From one side, the full hemisphere was obtained, and from the other side, the olfactory bulb and the hippocampus were removed.

### 4.5 Brain cryosectioning and immunocytochemistry

Fixed mouse and rat brains were stored in tissue-freezing medium (OCT embedding matrix, Leica Microsystems, Mannheim, Germany) at −80°C. The tissues were sectioned using a Leica Cryo-Microtome (Leica CM 1800, Leica, Germany). The cryo-sections were washed in PBS, and immunocytochemistry was then performed. The sections were cut at 12–14 µm thickness and then permeabilized using 1% Triton X-100 and 0.1% saponin (Sigma–Aldrich) dissolved in PBS. The sections were then blocked in 1% bovine serum albumin (BSA) in PBS for 1 hr. For immunocytochemistry, we used the following primary antibodies: anti-H3K9 acetyl (#06-942, Upstate-Millipore, Temecula, CA), anti-Sox2 (#ab7751, Abcam, Cambridge, UK), and anti-N-CAM (#701379, Thermoscientific, Rockford, IL). The samples were incubated with primary antibodies overnight at 4°C, washed three times in PBS for 5 min each, and then incubated with the appropriate secondary antibody at room temperature for 2 hr. The secondary antibody used was Alexa Fluor 594-conjugated donkey anti-rabbit IgG (#A21207, Life Technologies, Grand Island, NY), goat anti-rabbit IgG Alexa Fluor 488 (#ab150077, Abcam), and goat anti-mouse IgG Alexa Fluor 488 (#A21042). The primary antibodies were diluted 1:100, and the secondary antibody was diluted 1:200 in PBS containing 1% BSA. The nuclear DNA was counterstained using 4′,6-diamidino-2-phenylindole (DAPI; Sigma–Aldrich). The mounting medium was Vectashield (Vector Laboratories, Burlingame, CA). The mouse brain structures were identified in reference to the online Allen brain atlas (http://mouse.brain-map.org).

### 4.6 Confocal microscopy in tile-scanning mode was used to visualize brain sections

A Leica SP5-X laser-scanning confocal microscope (Leica Microsystems, Germany) was used to acquire images. To scan and analyze the interphase nuclei, we used Leica software (Leica LAS AF) with the following settings: 1024 × 1024 pixels, 400 Hz, zoom 8, and oil objective (HCX PL APO, 63×, NA = 1.4). The tile-scanning mode was used to visualize individual sections of whole brains. For image acquisition, we used the following objectives: 10× 0.25 DRY (N PLAN 1; Resolution yx: 781 nm, z-axe: 6916 nm; Leica Microsystems, Germany) or HCX PL APO lambda blue 20.0× 0.7 IMM UV (Leica Microsystems, Germany). Scanning was performed at a resolution of 512 × 512 pixels, and the acquired panels were auto-stitched using tile-scanning mode in smooth mode with a speed accuracy of slow/fine.

### 4.7 Western blot analysis

Western blot analysis was performed as previously described (Legartova et al., 2013). In the analysis, we used the following primary antibodies: anti-H3K9 acetyl (#06-942, Upstate-Millipore), anti-histone H3 (#ab1791, Abcam), anti-pan-acetylated-lysine (#ab-21623), anti-HDAC1 (#sc-7872, Santa Cruz Biotechnology, Santa Cruz, CA), anti-HDAC3 (#SAB1404635, Sigma Aldrich), and anti-H4ac (#382160, Merc Millipore, Germany). To evaluate the neuronal differentiation, we used anti-NCAM (#701379, Thermo-scientific), anti-Sox2 (#sc17320, Santa Cruz), anti-GFAP (#13-030, ThermoFisher Scientific), anti-GAPDH (#cs5174S, Cell Signaling), anti-p300 (#05-257 Merc Millipore, Czech Republic), and antibodies against βIII-tubulin (#T5076 Sigma-Aldrich) or Nestin (#ab6142, Abcam). We used the following secondary antibodies for Western blot analysis: anti-rabbit IgG (#A4914, Sigma Aldrich, Czech Republic), anti-mouse IgG1 (#sc-2060, Santa Cruz Biotechnology), and anti-mouse IgG (#A9044, Sigma Aldrich, Czech Republic). The total protein levels were measured using a μQuant spectrophotometer (BioTek Instruments, represented by Biotech, Czech Republic). The data for histone-associated markers were normalized to the total level of histone H3, while the levels of the neuronal markers were normalized to the total GAPDH level.

### 4.8 Chromatin immunoprecipitation (ChIP) combined with quantitative PCR (qPCR)

ChIP-qPCR was performed as previously described (Strašák et al., 2009) using a ChIP Assay Kit (#17-295; Millipore). The following antibodies were used: anti-H3K9ac (#06-942; Millipore) and anti-H4ac (#382160, Merck Millipore). As a negative control, we used samples incubated with anti-rabbit IgG (#A-4914; Sigma–Aldrich, Czech Republic). The primers for the Sox2 promoter and exon were as follows. Sox2 promotor: forward 5′-CCGCCGGAAACCCATT-3′ and reverse 5′-AGTTAATAGACAACCATCCATG TGATG-3′. Sox2 exon: forward 5′-ACTGCCCCTGTCGCACAT-3′ and reverse 5′-GAAAATCTCTCCCCTTCTCCAGTT-3′. GFAP promotor: forward 5′-AGCACCCCCATTGAATAGCC-3′ and reverse 5′-CTCAGTGGGGT-GAGAGGAGT-3′ (Sussman et al., 2013). Exon 6 of the housekeeping gene, GFAP: forward 5′-TGTCCAGGGCTAGCTTAACG-3′ and reverse 5′-CCAGTTACCAGGAGGCATTT-3′.

### 4.9 Software used for data analysis and statistical analysis

The following programs were used: Leica LAS AF software (Leica Microsystems), ImageJ software (see www.imagej.nih.gov, NIH USA freeware), and SigmaPlot software (version 13.0, Systat, San Jose, CA). With Sigma Plot software, we evaluated our data using paired Student’s *t*-test, and *p-*values ≤ 0.05 were considered as statistically significant. Statistically significant differences from control values are shown using asterisks (red asterisks show increased protein levels, and blue asterisks show decreased protein levels).

## Supplementary Material

Figure S1

Figure S2

Supplementary legends
