# Supplementary figures and images for: HDAC1 and HDAC3 underlie dynamic H3K9 acetylation during embryonic neurogenesis and in schizophrenia-like animals
Source: J Cell Physiol. Author manuscript; Available in PMC 2024 Apr 19. (PMC7615847; doi:10.1002/jcp.25914)

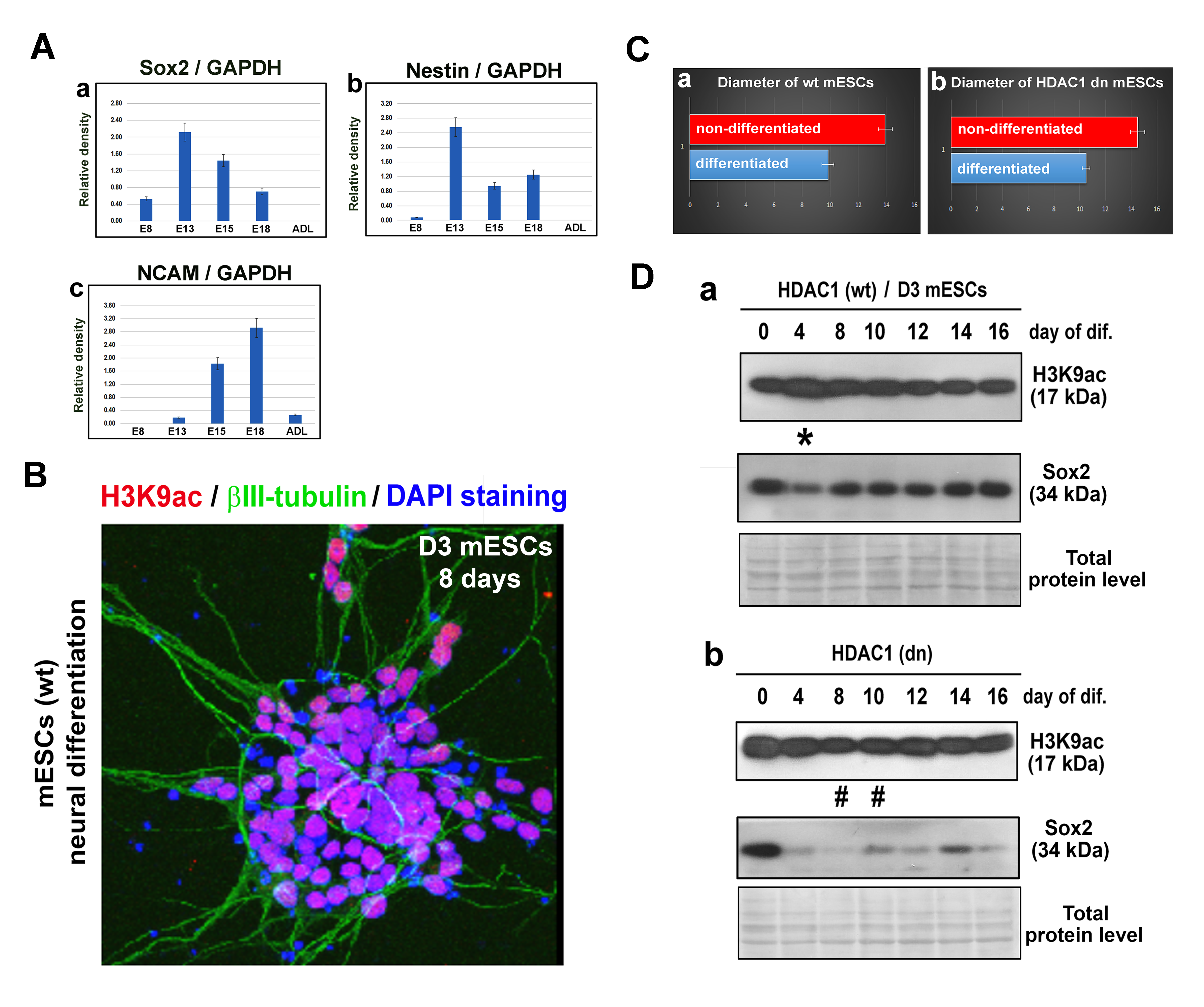

Supplement: Figure S1 [file EMS195214-supplement-Figure_S1.tif]

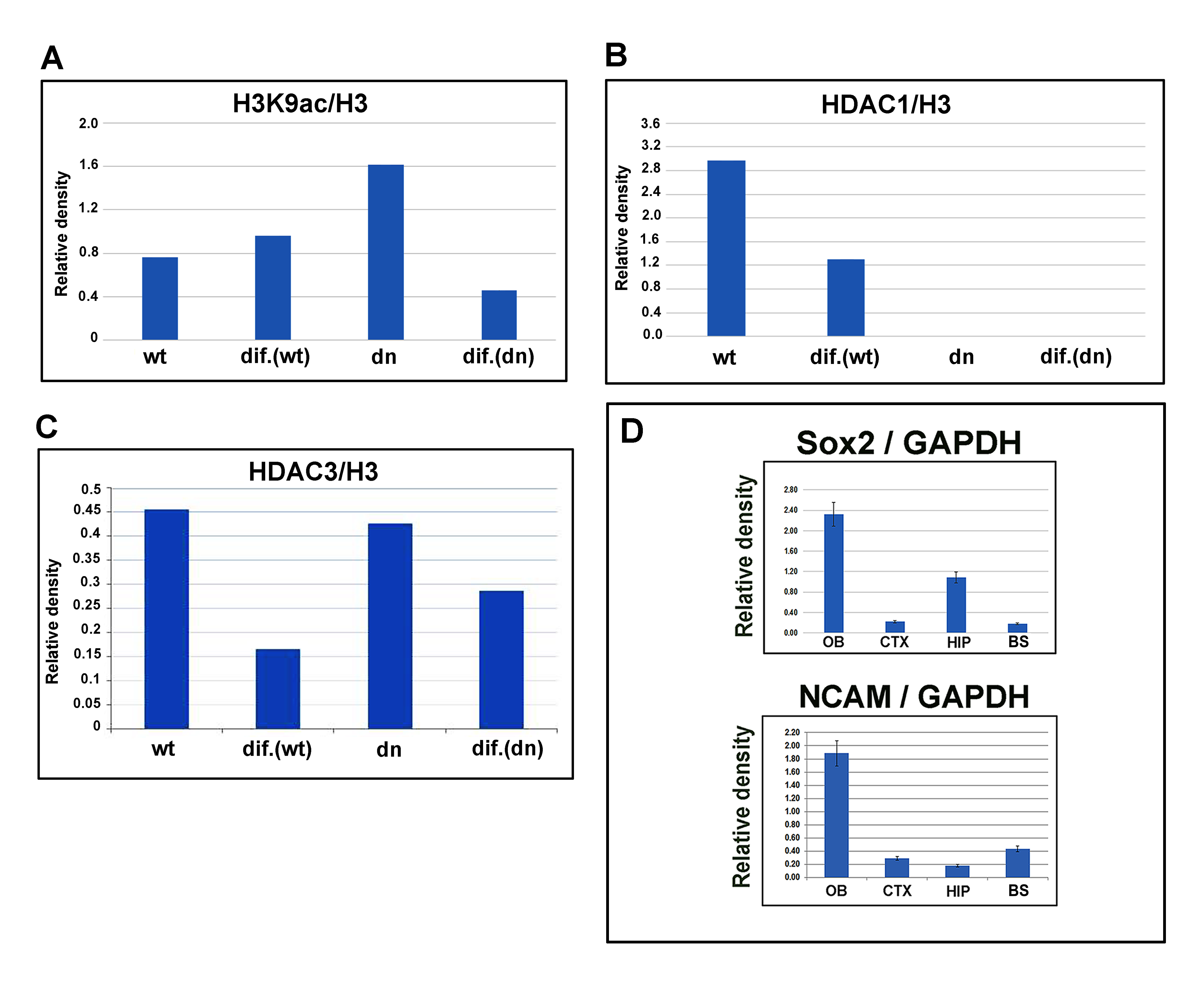

Supplement: Figure S2 [file EMS195214-supplement-Figure_S2.tif]
