# Supplementary material for: HDAC1 and HDAC3 underlie dynamic H3K9 acetylation during embryonic neurogenesis and in schizophrenia-like animals
Source: J Cell Physiol. Author manuscript; Available in PMC 2024 Apr 19. (PMC7615847; doi:10.1002/jcp.25914)
Supplement: Supplementary legends [file EMS195214-supplement-Supplementary_legends.docx]

**Supporting legends:**

**Supplementary Fig. 1.** (*A*) Quantification of the western blot data shown in Fig. 1E for the (*a*) Sox2, (*b*) Nestin, and (*c*) NCAM proteins. The data were normalized to the level of GAPDH. (*B*) Experiments performed using immunofluorescence on the 8^th^ day of neural differentiation in induced (wt) D3 mESCs. H3K9ac (red) was observed in the center of the colonies, and βIII-tubulin positivity was observed in the more peripherally positioned mESCs, which lacked H3K9ac. (*C*) Comparison of the diameters of non-differentiated and differentiated (*a*) wild-type mESCs and (*b*) HDAC1 (dn) mESCs. (*D*) Western blot analysis of H3K9ac in (*a*) (wt) mESCs and (*b*) HDAC1 (dn) mESCs that had undergone neural differentiation for 16 days. The densities of the western blot bands showing H3K9ac were normalized to the total protein levels. For the individual experimental conditions, the levels of Sox2 are documented. The asterisk in panel D shows the highest level of H3K9ac, which was observed in the (wt) cells, and the bullets show the lowest level of H3K9ac, which was observed in the HDAC1 (dn) mESCs.

**Supplementary Fig. 2.** Quantification of the western blot data shown in Fig. 4Ca. The panels show the levels of (*A*) H3K9ac, (*B*) HDAC1, and (*C*) HDAC3, each normalized to the levels of histone H3. The analysis was performed in non-differentiated and differentiated (wt) and HDAC1 (dn) mESCs. (*D*) Quantification of the western blot data shown in Fig. 5D demonstrating the levels of Sox2 and NCAM in the olfactory bulbs (OB), brain cortex (CTX), hippocampus (HIP) and brain stem (BS).
